# Supplementary material for: Patterns of Intron Gain and Loss in Fungi
Source: PLoS Biol. 2004 Nov 30;2(12):e422. doi: 10.1371/journal.pbio.0020422 (PMC532390; doi:10.1371/journal.pbio.0020422)
Supplement: Table S1 — Also available at http://genes.mit.edu/NielsenEtAl/. (4.3 MB ZIP). [file pbio.0020422.st001.zip › NielsenEtAl/html/1187.html]

AN5011.1.NCU05895.1.MG03376.1.FG06069.1


```
 CLUSTAL W (1.82) Multiple Sequence Alignments - Introns Inserted


Sequence 1: NCU05895.1	994 aa
Sequence 2: MG03376.1	964 aa
Sequence 3: FG06069.1	961 aa
Sequence 4: AN5011.1	949 aa
Alignment Length: 1007 aa
Number Identitical Residues: 478 aa
Alignment Score (without introns) 24542


MG03376.1 	MASRLHRPG-----IG~PTGCDMTVAQLDLAQQ~LATEALPIG------Q0IVQNEWYGY
NCU05895.1	MASSRYKDKDAGVVLS~FNGQWISWTHTVVAYA1AFLSALIIGCALHYHK~IVKNEWYGY
FG06069.1 	MAS-KYKDKDTGVVLS~FNGSLVSWAHTTVAYT1AFFSALVIGLYLHYHK~IVQNEFYGY
AN5011.1  	MAP-KFKDGD--AVAT0LNGKWVSWAHTAVAYT~AFLSALFVGMSLHFNK~IVQNEHYGY
          	**.  .:  .        .*  :: ::  :*      .** :*      : **:** ***

MG03376.1 	PQEWFPSVSATIGDKYPERSVFMLLIAITSV1LTTQLRIG~PRFALVGLWYLLTRRNGQA
NCU05895.1	PDEWFPSVSATIGDRYPERSIFMIFIAITS-~-------G1PRFALVGLWYLLTAKPGRV
FG06069.1 	PQEWFPSVSATIGDRYPERSFFMIFIAITS-~-------G1PRFALVGLWYLLTRKPGQK
AN5011.1  	PDEWFPSVSATIGDRYPERSFFQVFIAITS-~-------G~PRFALVFLWYLVTARPNST
          	*:************:*****.* ::*****         * ****** ****:* : .  

MG03376.1 	LPKFVAITGVVRTLTCGGWTYITSTDDHDWHDILMISYIVATIPWTAGCIALSPPNAKAI
NCU05895.1	LPKAIAISGVIRTLTCGGWTYITSTDDHDWHDILMILYIVFTIPWTTGCIALSPPNAKAI
FG06069.1 	LPTFVAVMGLVRTLTCGGWTYITSTDDHDWHDILMISYIVATLPWTTGCIVLSPANPQAI
AN5011.1  	LPKFVAGVGIFRTFTCGGWTYVTSTDDHDWHDIFMISYLVATLPWTIGCLALSPNNRRAV
          	**. :*  *:.**:*******:***********:** *:* *:*** **:.*** * :*:

MG03376.1 	KYRKYLCSAFFGTLVPLIYFFIQHKVHRVAG--------------------------A1Y
NCU05895.1	KYRKWIAGSFFGTLVPLVYFFIQHKVHRVAGGRFPVLEMLLRQTQYNVLTFAPMRRTA~Y
FG06069.1 	KYRKYLASAFFGTLVPLIYFFIQHKVHRVAG--------------------------A1Y
AN5011.1  	KYRKILASLFFGTLVPLIYYFIQHKVHKVPG--------------------------A1Y
          	**** :.. ********:*:*******:*.*                          * *

MG03376.1 	TIYAFFEWSLILFDVGFDAVTALDFDSFELIIKDVHGASQG2TNVSPQKAAVLEKE2KEK
NCU05895.1	TVYAFFEWALILFDVAFDAVTALDFDTFEVVVKDVKGASKG2ANASSVPAAVLEKE2KEK
FG06069.1 	TTYAFFEWSLIIFDVAFDAVTALDFSTFDIEVRDVRGARKG2ENHSSVPSAVLEKE2KEK
AN5011.1  	TRYAFFEWSLILFDVGFDAVTALDFEAFEIVVKDVKGISRG2Q-LKNTADSVLEKE2KGK
          	* ******:**:***.*********.:*:: ::**:*  :*    .    :***** * *

MG03376.1 	ATAGLMHQKVTPSGMLDIAADVYHG0FVFWSILTSLGVTIWY1FPLWYMGISGYEALVMV
NCU05895.1	ATAGVYSAGFNLGPALDIAADVYHG0FVFWSTLTSLGLVIWY1FPLWHMGISGYEAFVMA
FG06069.1 	ETGGVLAVRFSWSEALDIAAEIYHG0YVFWSILTSLGLVVWY1FPLWYMGISGYEVSVMT
AN5011.1  	PVGNTFGQGFFWSEVFDAAADVYNG0FVYWTNWTGLPVLVWY~FPLWHMGISGYEVVILS
          	 ...     .  .  :* **::*:* :*:*:  *.* : :** ****:*******. :: 

MG03376.1 	SISPLLLGISPFRWLITRFQRFFHLLSLTGLVAFLIKDPIQRLYAVGFGLGMQCLAWVAT
NCU05895.1	TISPVLLGIRPLRSLIVNNQRLFHVLSLAGLLAYKVHDPVYRLFTVGFGVFMGCLAWAAT
FG06069.1 	TISPFILASRSARSLVVNNLRVVHLLSLAGIAAYLVEEPTMRLFSVGFGVFMSCLGWAGT
AN5011.1  	YVAPVLLAIPALKSLAIKNPRIFHFLSLSGFLAYKVQNPANRLFVSTFAVACSSITFAAT
          	 ::*.:*.  . : *  .  *..*.***:*: *: :.:*  **:   *.:   .: :..*

MG03376.1 	LHSESVHEGRLELRILSWAVGLILSSTVKFACRTLNPIWPIMRAETGGWNYTGLFLGLIA
NCU05895.1	WSSDSVHPSRLESRVIAWLAGLLMSSIAKFAWYTNNPIWPVMHAENGGWNGTGLVLAILA
FG06069.1 	LFAESVHEGRLESKILGWMIGLILSSTAKFAWWTNNPIWPIMHAANGGWNNTGLVLGIIA
AN5011.1  	LYGDRAQHTKLESRVFAWGLGLILSSIAKFAGKTNNPFWPIMHAENGGWNKLGLLLAILA
          	  .: .:  :** :::.*  **::** .***  * **:**:*:* .****  **.*.::*

MG03376.1 	AWR-FTRRGPLNLDGV--QIKSGSSFLAACSIGGLFFALHSLLSDTSTMILWVWDGYPIR
NCU05895.1	VLR-FTRKAPLNSWGVS-QQKNGSSLLSAIGIGGLFFAIHSLLSDTSTMILWVWGGFPVR
FG06069.1 	ALR-FTRRTPLTAGLVTSPAKTSSSFLASLGLAGLFFGLHSLLSDTSTMILWGWEGYPIR
AN5011.1  	GIRSFRVPASSGGDYFPSTGKKGSSLLAGLGLGSMIFLMHYLLSDSSTMIAWVWEGYPVR
          	  *:*    .     ..:  *..**:*:. .:..::* :* ****:**** * * *:*:*

MG03376.1 	GPTSNTTAFYTIFAMTAGVLIGAYKPGLASLWTSYFIGCVGAGMVTIYPHWKGYYGALAL
NCU05895.1	GPVSNVHAYYTIAAMTGGLLIGVFRPGMITNWTAYGVACVGAAMLTLYEQWFGYYGALIL
FG06069.1 	GPYFSTHGWLTVLAMSLGVFSGVWQPRVASSWGAYIIGTAGAMFLTFFSHWSGYYGALTL
AN5011.1  	GPIAVPHGALTIFAMGAGLAFGLFYPNVAGSWTAFGIGSVGAAFLTGTSHWSGFYGALTI
          	**     .  *: **  *:  * : * :   * :: :. .** ::*   :* *:**** :

MG03376.1 	TTYLMAVAVPLLRDAAKRSPGLVFGLGFFIYNFMVLFHVWVVAYAFVPGGPLVREHTDWV
NCU05895.1	TTYLMAIAVPMISKAAKKNPAVTFGVGFLFYNLLVLFHVWVVAYAFVPGGPYVREHTDWI
FG06069.1 	ATYLMAYSVPILVHAAKTSPATTFGNGFLIYNFLVLFHVWVVAYAFVPGGQLVREHTDWV
AN5011.1  	SFYLMAVSPVMISSAVRHSPAITFGLAFFVYVFLLLFHVWVVAYAFVPGGPLVREHTDWL
          	: **** :  ::  *.: .*. .** .*:.* :::***************  *******:

MG03376.1 	MASMMVLIGCGVFNLNAGKAAKKSKGKNEAQTSRSTLQHRKHYITVLGAINVAFLVAAYL
NCU05895.1	MLTTMLTIGAGVFDLVSGQANHKYAASTDRRSS--PLGHRKYHVGILGLLNILFLVANFL
FG06069.1 	MYTMMTCIGAGVYGINVSSPQRQTPKRS------LPTQQRKLFGISTIMINILFLISAFQ
AN5011.1  	MTVAMLFIGAGVFSAAVSRSSGPRTNAISPNGR----RLRSYYVYVLVALQLLSVSIAYL
          	*   *  **.**:.   . .        .          *. .      :::  :   : 

MG03376.1 	RFPTNDYKPYHAPDRVITAGMWTIHFGLDNDMWSSERRMRDLLRDAELDVIGLVESDQQR
NCU05895.1	RFPSYDYKPYHAEDRLFTAGIWTIHFSLDNDMWSSEYRMRDLIKELEVDVIGLLESDLQR
FG06069.1 	RFPANNHRPYHGEDRILTAGIWTIHFSFDNDMWASEYRMRDLIKELEIDVIGLLESDNQR
AN5011.1  	RFPTNDYTPYHKEEKAVTAGIWTVHFGLDNDMWASERRMRDVIQELEIDVLGLLESDNQR
          	***: :: ***  :: .***:**:**.:*****:** ****:::: *:**:**:*** **

MG03376.1 	IIMGNRDSTQYLAEDLGMYVDYGPGPNKHTWGAALLSKFPIINSTHHLLPSPVGELAPAI
NCU05895.1	IIMGNRDTTQFLAEDLGMYVDYGPGPNKHTWGAALLSKFPILNSTHHLLPSPVGELAPAI
FG06069.1 	IIMGNRDATQFLAEDLGMYVDYGPGPNKHTWGAALLSKFPIMNSTHHLLPSPVGELAPAI
AN5011.1  	IIMGNRDITQFIAEDLGMYADFGPGPNKHTWGSALLSKFPIVNSTHHLLPSPVGELAPAI
          	******* **::*******.*:**********:********:******************

MG03376.1 	HATLDVYGTMVDVFVFHSGQEEDPEDRRLQSEYMRELMGST--NRPSVLLSYLVTRPLEG
NCU05895.1	HATLDVYGSLVDVFVFHSGQEEDPEDRRLQSEYLAKLMGSIPADRPALLLSYLVTKPLEG
FG06069.1 	HATLDVYGQPVDVFVFHSGQEEDPEDRRLQSLYLAELMGST--PRPAFLLSYLVTKPKEG
AN5011.1  	HATLDMYGELVDVVVFHSGQEEDPEDRRLQTEYLSKLMGSS--PRPLILLSYLVTKPLEG
          	*****:**  ***.****************: *: :****    ** .*******:* **

MG03376.1 	NYNTYVSEQSGMHDVDPTDWDRWCEYILFKGLKRVGYARISRSTITDTELQ~VGKFVVPE
NCU05895.1	NYNTYVSDISGMHDVDYTDWDRWCEYILYKNLLRTGYARVSRSTITDTELQ~VAKFIVPK
FG06069.1 	NYNTYVSDKSGMKDVDPSDWDRWCEYILFKRLKRVGYARVSRSSITDTELQ~VAKFKIPE
AN5011.1  	NYNTYVSELSGMKDIDQTDWDRWCEYILYKNLKRTGYARVSRDSITDTEIQ0VGKFVIGE
          	*******: ***:*:* :**********:* * *.****:**.:*****:* *.** : :

MG03376.1 	PGSAEATRAWEVDAEARNSRVEESQVPEGLRFPAVFRGEGVRGHRYHVFDEPR~YFNY-
NCU05895.1	-DDADKGRILAASKEQRDRRVEEQQVPDGWRFPALFRGEGVRGHHYHVFDEPR~YYFYF
FG06069.1 	-SKEEIAKLEAQSDKERNRQVQEKDVPEGWRFPAMFRGDGVRQHRYHVFNEPR2VGDV-
AN5011.1  	------------PEPENEMMIPEEMVPQGLRFPAMFRGQGVRGHRYHVFNEPR~YWQ--
          	                .:  : *. **:* ****:***:*** *:****:***
```
